# Factors Related to Bone Metabolism in Kidney Transplant Recipients

**DOI:** 10.1155/2021/6679095

**Published:** 2021-01-13

**Authors:** Chenxiu Wang, Yanan Huo, Xinchang Li, Anhua Lin, Qingxiang Hu, Changhui Xiong, Ying Deng

**Affiliations:** ^1^Department of Endocrinology, Jiangxi Provincial People's Hospital Affiliated with Nanchang University, Nanchang, China; ^2^Department of Transplantation, Jiangxi Provincial People's Hospital Affiliated with Nanchang University, Nanchang, China; ^3^Department of Science and Education, Jiangxi Provincial People's Hospital Affiliated with Nanchang University, Nanchang, China

## Abstract

This study is aimed at establishing the prevalence of osteoporosis and osteopenia in kidney transplant recipients (KTRs) and determining the risk factors for bone mass loss. We invited KTRs who were under regular follow-up at Jiangxi Provincial People's Hospital Affiliated with Nanchang University to attend an assessment of osteoporotic risk assessed by questionnaire, biochemical profile, and dual-energy X-ray absorptiometry (DXA) scanning of the lumbar spine, total hip, and femoral neck. Binary logistic regression models were used to investigate the relationship between the different variables and bone mass density (BMD). A total of 216 patients satisfied the inclusion criteria. The group consisted of 156 men (72.22%) and 60 women (27.78%), and the mean age was 41.50 ± 9.98 years. There were 81 patients with normal bone mass (37.50%) and 135 patients with bone mass loss (62.50%). Logistic regression analysis showed that a higher phosphorus value and higher alkaline phosphatase concentration and a longer use of glucocorticoids were risk factors for bone mass loss in KTRs, and maintaining an appropriate weight and exercising an appropriate number of times per week helped to maintain bone mass.

## 1. Introduction

Kidney transplantation is a common and effective treatment modality for end-stage renal failure. Successful transplantation is capable of reversing many complications of renal failure; however, disturbances in bone and mineral metabolism may persist and be associated with a high risk of fracture, morbidity, and mortality. Kidney transplant recipients (KTRs) are known to have an increased risk of bone loss, and fracture risk is also higher than those for the general population and patients on dialysis [1–4]. At present, the focus of kidney transplantation research is mainly on the maintenance of renal function after transplantation and the side effects of immunosuppressive agents. The prevalence of osteoporosis of KTRs is estimated to be close to 30% [3], and an estimated 22.5% of patients will experience a fracture within the first 5 years following transplantation [4]. Intuitively, any treatment intervention to preserve bone mass density (BMD) in KTRs should be directed at the underlying cause; thus, identifying the risk factors for this complex pathophysiological situation is an attractive proposition. Bone loss after renal transplantation has not been well quantified in KTRs nor have the factors that may contribute to bone loss in this population been well elucidated. Therefore, this cross-sectional study was designed to establish the prevalence of bone loss and osteoporotic fractures and evaluate the risk factors for bone health in KTRs.

## 2. Materials and Methods

### 2.1. Patients

We invited 234 KTRs who were under regular follow-up by Jiangxi Provincial People's Hospital Affiliated with Nanchang University to attend an assessment of osteoporotic risk from August 16, 2018, to September 16, 2019. Exclusion criteria included systemic illness, prolonged immobilization, liver disease, Cushing syndrome, and chronic gastrointestinal disease (chronic diarrhea or malabsorption). Patients with a history of thyroid disease before or after transplantation (hyperthyroidism or hypothyroidism) were excluded. The inclusion criteria included age 18 years or older, completed kidney transplant, and sign an informed consent. A total of 216 patients did not meet the exclusion criteria and met the inclusion criteria.

### 2.2. Methods

Bone health risk was assessed by questionnaire, biochemical profile, and dual-energy X-ray absorptiometry (DXA) scanning at the lumbar spine, total hip, and femoral neck. The contents of the questionnaire include age, sex, education, marriage, hemodialysis duration, age at the start of dialysis, age at transplantation, age of menopause, milk intake, exercise sessions per week, smoking habit, time outdoors, alcohol abuse, fracture, reason for the renal failure, renal source and duration of glucocorticoid use (glucocorticoids are converted to prednisone), average daily glucocorticoid dose, duration of cyclosporine, duration of tacrolimus, duration of mycophenolate mofetil (MMF), and exogenous intake of vitamin D and calcium (pre- and posttransplantation). Height and weight were measured, and the BMI was calculated.

Cumulative doses of glucocorticoids were calculated from outpatient and inpatient case data records, which included pulsed doses of intravenous methylprednisolone given during transplant rejection episodes. However, other immunosuppressants were given in varied doses according to the concentration of the drug. Thus, it is difficult to calculate the cumulative exposure, so the variable we use is the duration of use. Smoking history was defined as continuous or cumulative smoking for 6 months or more, and alcohol abuse was defined as an average daily alcohol intake ≥3 U (1 U≈285 ml standard beer/30 ml liquor/120 ml wine). Fracture information was obtained from the medical history and thoracolumbar anterolateral radiographs.

Routine laboratory tests including creatinine (CR), albumin (ALB), serum calcium, phosphorus, alkaline phosphatase (ALP), blood urea nitrogen (BUN), carbon dioxide combining power (CO2CP), total cholesterol (TC), triacylglycerol (TG), high-density lipoprotein cholesterol (HDL-C), low-density lipoprotein cholesterol (LDL-C), and fasting blood glucose (FBG) were measured using an automated multichannel analyzer (Olympus AU 800 automated multichannel analyzer, UK). Serum intact parathyroid hormone (iPTH), 25(OH)D (25-hydroxyvitamin D3), N-terminal propeptide of type 1 collagen (P1NP), and *β*-isomerized C-terminal telopeptide of type 1 collagen (*β*-CTX) were assessed by an automatic electrochemical luminescent immunoassay (Roche Cobas e601 automatic electrochemical luminescent immunoassay system, Switzerland).

BMD measured in grams per square centimeter was determined using DXA (Hologic Discovery 89098 densitometer, Waltham, MA, USA) for the lumbar spine (L1-L4 in the anteroposterior direction), total hip, and femoral neck. BMD was expressed in standard deviation units as *t* scores (comparison with the young adult mean) or as *z* scores (comparison with the age-matched mean). The following are the scores according to the diagnostic criteria of osteoporosis published by the World Health Organization (WHO) in 1994 [5]: postmenopausal women and men over 50 years old: *t* value ≥-1.0 SD indicates normal bone mass, a *t* value between -1.0 and -2.5 indicates osteopenia, and a *t* value ≤-2.5 indicates osteoporosis; premenopausal women and men younger than 50 years of age: *z* value ≤-2.0 is “below the expected range for age”, and *z* value >-2.0 is “within the expected range for age”. In addition, osteoporosis is also diagnosed in patients with brittle fractures. To facilitate the study, osteopenia, osteoporosis, and “below the expected range for age” are collectively associated with “bone mass loss”, and the rest of the population has “normal bone mass.”

### 2.3. Statistical Analysis

Demographics and other characteristics were summarized using descriptive statistics with continuous variables that were normally distributed and reported as the mean ± standard deviation (SD), and categorical variables are presented as numbers (percentage) or for nonparametric data as the median (interquartile range). Continuous variables were compared using Student's *t*-tests, the statistical verification value was expressed as *t*, and a *p* value of less than 0.05 was considered statistically significant. Pearson's *χ*^2^ test or Fisher's exact test was used to compare categorical variables, the statistical verification value was expressed as *χ*^2^, and a *p* value of less than 0.05 was considered statistically significant. Ordinal data were compared using the Mann-Whitney *U* test, the statistical verification value was expressed as *z*, and a *p* value of less than 0.05 was considered statistically significant. Univariate variables were included in the binary logistic regression analysis model, and stepwise regression analysis was used. A *p* value of less than 0.05 was considered statistically significant. All statistical analyses were performed using SPSS version 23.0 (Version 22; SPSS Inc., Chicago).

### 2.4. Informed Consent Was Obtained from all Patients, and the Local Ethics Committee Granted the Approval

## 3. Results

### 3.1. Descriptive Characteristic

A total of 216 patients satisfied the inclusion criteria. The group consisted of 156 men (72.22%) and 60 women (27.78%), among whom 20 (9.26%) were postmenopausal and 31 (14.35%) men were aged 50 or older. The mean age was 41.50 ± 9.98 years. We divided the patients into two groups: 81 patients with normal bone mass (37.50%) and 135 patients with bone mass loss (62.40%). Of the 20 postmenopausal women, 9 had osteoporosis, and 8 had osteopenia, which combined accounted for 85% of these postmenopausal participants. Among the 40 premenopausal women, 19 patients had bone mass loss, accounting for 47.50%. There were 7 cases (3.24%) with a history of brittle fracture. The anatomic fracture sites were as follows: including the spine (*n* = 2), forearm (*n* = 2), leg (*n* = 2), rib (*n* = 1), and hip (*n* = 1), ages ranged from 36-58 years, and the mean age was 47.43 ± 8.56 years. Seventy patients (32.41%) had smoking habits, and 41 patients (18.98%) abused alcohol. There were 15 KTRs (6.94%) who had diabetes listed as one of the diagnoses (among them, 4 patients had a history of diabetes, and 11 patients had fasting blood glucose greater than 7 mmol/L by monitoring, where postprandial blood glucose was not detected for 2 hours), and 8 of those were diagnosed with bone mass loss.

The cause of end-stage renal failure was glomerulonephritis in 68 patients, polycystic kidney in 4, hypertensive nephropathy in 25, diabetic nephropathy in 2, IgA nephropathy in 22, systemic lupus erythematosus in 1, and gouty nephropathy in 3, and in 87 cases, the cause was unknown or missing. Almost all kidney transplant recipients had been on long-term dialysis pretransplantation (214/216 99.07%). Patients underwent dialysis for a median of 19.04 months before transplantation. Most of the kidney supply is from unrelated donors (180/216, 83.33%).

None of the recipients had ever been treated with bisphosphonates, denosumab, calcitonin, or teriparatide. Seventy-one patients were taking vitamin D agents or calcium agents (32.87%) before transplantation and 9 patients after transplantation (4.17%). All subjects had received immunosuppression treatment with glucocorticoids, tacrolimus, MMF, or cyclosporine. Among the 216 patients, a serum calcium level higher than 3 mmol/L was rarely observed; hypercalcemic episodes (defined as total serum calcium > 2.62 mmol/L) were reported in 19 and 8.79% of KTRs, whereas hypophosphatemia (phosphorus < 2.5 mg/dl) was reported in 8 and 3.70% of KTRs. We defined insufficiency as 25 − OHD < 30 ng/ml, and it was reported in 100 and 46.30%; deficiency as 25 − OHD < 20 ng/ml, which was reported in 61 and 28.24%; and severe deficiency as 25 − OHD < 10 ng/ml, which was reported in 9 and 4.17%. More KTRs were in CKD stages 1 and 2, and fewer were in stages 3-5.

The baseline demographic, anthropometric, and lifestyle variables of the patients are presented in Table 1. There were statistically significant differences in weight, age, BMI, and exercise sessions per week between the low bone density group and the normal bone density group (*p* < 0.05).

The biochemical characteristics of the patients are presented in Table 2. There were statistically significant differences in phosphorus levels between the low bone density group and the normal bone density group (*p* < 0.05).

All patients were treated with glucocorticoid, tacrolimus, and cyclosporine or glucocorticoid, tacrolimus, and MMF immunosuppressive therapy after transplantation.

The use of immunosuppressive drugs in the two groups of patients is shown in Table 3. There were statistically significant differences in the duration of glucocorticoids, cumulative glucocorticoids, duration of cyclosporine, and duration of MMF between the low bone density group and the normal bone density group (*p* < 0.05).

### 3.2. Binary Logistic Regression

The two groups of statistically significant variables were included in the binary logarithmic regression model in the above three tables, and logistic regression analysis showed that phosphorus, alkaline phosphatase, BMI, exercise sessions per week, and the duration of glucocorticoids were the factors that truly affected the BMD of KTRs. Among them, phosphorus, alkaline phosphatase, and the duration of glucocorticoid use were the risk factors affecting bone mineral density. BMI and weekly exercise sessions were protective factors that affected bone mineral density (Table 4).

## 4. Discussion

The decrease in BMD measured by DXA occurs in the first 12 months after transplantation and seems to slow down thereafter but at significantly lower levels than in healthy controls [6]. The incidence of bone mass loss was found to be 62.4% based on our previously defined criteria, which is consistent with most studies so far. The prevalence of bone mass loss in KTRs is higher, which indicates that while paying attention to cardiovascular disease and transplant function in kidney transplant patients, bone metabolism should also be considered. A systematic literature review by Naylor et al. found that fracture rates ranged from 3.3 to 99.6 fractures per 1,000 person-years [7]. The overall fracture risk after renal transplantation is 3.6–3.8-fold higher than that in healthy individuals and is 30% higher during the first 3 years after transplantation than that in patients before transplantation [8, 9]. However, to our surprise, the prevalence of fractures was not as high as we thought; the prevalence of fracture was only 3.24% in our population. The differences may be due to the different definitions of fractures used and the different characteristics of the population. However, overall, this group will have a higher fracture risk than the general population, leading to an associated increase in morbidity and mortality.

Our studies showed an independent association between exercise sessions per week, BMI, and bone mass in KTRs. This suggests that maintaining an appropriate weight and weekly exercise routine may have positive implications for maintaining bone mass in KTRs. We found that smoking and milk intake do not affect bone health as much, and it may take a longer time to observe and require a larger sample size to discover their effects on bone mass and fractures. The duration after transplantation and duration of dialysis in KTRs also did not affect bone mass. A prior study demonstrated that BMD increased or remained stable several years after transplantation [10]. In our study, we did not observe a relationship between age, sex, and bone mass of KTRs, probably because the average age of the study population is young, and most of them were premenopausal women and mature men.

Abnormal phosphorus and calcium concentrations are common and fluctuate widely in the first year after kidney transplantation. Therefore, the KDIGO 2017 guideline update recommends that serum calcium and phosphorus levels be measured at least weekly in the immediate post-kidney transplant period until stable [11]. In our study, hyperphosphatemia was a significant factor for the negative effect on bone density. Hyperphosphatemia is usually seen in patients with delayed graft function or in patients with advanced CKD. For patients with CKD G3a–G5D, the 2019 Chinese Guidelines suggest lowering elevated phosphate levels toward the normal range. For patients whose serum phosphorus exceeds the target value, the guidelines suggest reducing dietary phosphorus intake (800-1,000 mg/day) alone or in combination with other phosphorus reduction treatments [12]. No correlation was found between calcium and low bone density.

Vitamin D insufficiency and deficiency are extremely widespread among KTRs. The reported prevalence of vitamin D insufficiency after transplantation is 51-97%, and deficiency is 26%-33% [13, 14]. Our data were similar; vitamin D insufficiency accounted for 74.5%, and deficiency accounted for 28.2%, much higher than those who did not undergo kidney transplantation. Most patients in this study were not supplemented with vitamin D and its derivatives. Different regions, different populations, and different seasons are also important factors that determine the vitamin D status of KTRs.

The study found that increased ALP is an independent risk factor for low bone mass. Elevated ALP indicates a high conversion state of bone, and the rate of bone loss is accelerated. Our study did not find a relationship between iPTH and bone mass loss. The association between high iPTH levels and low bone mass might be mainly prevalent in short-term transplant patients. Over the very long term after transplantation, iPTH levels decrease and lose an association with BMD. Bone biochemical indicators such as PINP and *β*-CTX were found to have nothing to do with bone density.

Glucocorticoids are commonly prescribed for KTRs and have a profound inhibitory effect on bone formation by targeting osteoblast proliferation and differentiation while stimulating apoptosis of osteoblasts and osteocytes, thereby reducing bone turnover and synthesis [15]. In addition, glucocorticoids influence the synthesis of IGF-1, an osteoblast activator, by inhibiting IGF-1 gene transcription [16]. In our population, all KTRs were prescribed with glucocorticoids, and focusing on the duration of glucocorticoid use may show the true relationship between hormones and bone density. Early glucocorticoid withdrawal has been associated with a significant reduction (31%) in fracture risk and fracture-induced hospitalization among patients [17]. We also found that the longer the use of glucocorticoids, the lower was the bone density. However, the cumulative, current, and average daily dosage of glucocorticoids in our patients had no association with bone mass loss. In addition, BMD [18, 19] and fractures [20] decrease with prednisolone-sparing, prednisolone-withdrawal, and prednisolone-limiting protocols. The skeletal effects of other immunosuppressive agents remain uncertain.

Supplementation with vitamin D has been shown to improve components of mineral and bone disease, such as reduced PTH levels and possibly improved bone mineral density. Josephson et al. found that treatment with calcitriol (0.25 *μ*g/d) and calcium (1 g/d) led to a significant 4.8% gain in FN-BMD after 12 months and unchanged LS-BMD [21]. In our study, the number of cases of vitamin D supplementation before and after transplantation was too small to determine the relationship between bone mass losses. Most recommend the use of an adequate dose of vitamin D to correct vitamin deficiency and maintain a serum 25(OH)D level of >30 ng/ml.

Management of posttransplant bone disease is challenging. This study found that the special population of KTRs has a very high incidence of low bone mass, which is higher than the general population of the same age. With increasing age, the incidence of osteoporosis and fracture in this population will further increase, bringing a large economic burden to families and society. Reducing the time of steroid exposure, maintaining an appropriate body weight and the number of weekly exercise sessions, and correcting abnormal phosphorus metabolism may help maintain the bone mass of KTRs. The contribution of this study is that these people have hardly received any medical intervention for the prevention and treatment of osteoporosis, which shows the natural changes in the bone health status of KTRs. Our study has some limitations. First, it was conducted in a single center and is a cross-sectional study that does not truly reveal the causal relationship between variables and bone mass loss. Due to the small number of fracture events, we cannot determine whether there is a correlation between the changes in bone density and fractures.

## Figures and Tables

**Table 1:** Baseline demographic, anthropometric, and lifestyle variables of the patients.

	Normal bone density (*n* = 81)	Low bone density (*n* = 135)	*t*/*χ*^2^/*z*	*p*
Height^∗^	162.57 ± 6.54	162.12 ± 7.08	*t* = 0.465	0.643
Weight^∗^	59.65 ± 9.59	56.52 ± 9.93	*t* = 2.276	0.024
Age at transplantation^∗^	37.84 ± 7.72	36.84 ± 10.49	*t* = 0.805	0.422
BMI^∗^	22.52 ± 3.04	21.44 ± 3.19	*t* = 2.444	0.015
Sex^†^			*χ* ^2^ = 0.190	0.663
Male	57 (36)	98 (63.2)		
Female	24 (40)	36 (60.0)		
Age^†^			*χ* ^2^ = 27.882	≤0.001
18-29	8 (30.8)	18 (69.2)		
30-39	20 (32.3)	42 (67.7)		
40-49	48 (57.1)	36 (42.9)		
≥50	5 (11.4)	39 (88.6)		
Education^†^			*χ* ^2^ = 1.691	0.429
Illiteracy-primary	10 (45.5)	12 (54.5)		
Middle school, high school, or technical secondary school	55 (38.7)	87 (61.3)		
College and above	16 (30.0)	36 (69.2)		
Marriage^†^			*χ* ^2^ = 3.437	0.064
Married	74 (40.0)	111 (60.0)		
Unmarried	7 (22.6)	24 (77.4)		
Milk intake^†^			*χ* ^2^ = 2.670	0.104
Yes	20 (48.8)	21 (51.2)		
No	61 (35.1)	113 (64.9)		
Outdoor time^†^			*χ* ^2^ = 0.772	0.441
<30 min	14 (32.6)	29 (67.4)		
≥30 min	67 (39.0)	105 (61.0)		
Exercises sessions per week^†^			*χ* ^2^ = 2.124	0.035
Seldom	29 (29.9)	68 (70.1)		
1 to 4 times a week	15 (41.7)	21 (58.3)		
More than five times a week	37 (45.1)	45 (54.9)		
Smoking habit^†^			*χ* ^2^ = 0.035	0.852
Yes	27 (38.6)	43 (61.4)		
No	54 (37.0)	92 (63.0)		
Alcohol abuse^†^			*χ* ^2^ = 1.119	0.291
Yes	18 (43.9)	23 (56.1)		
No	63 (36.0)	112 (64.0)		
Duration after transplantation^†^			*χ* ^2^ = 4.614	0.033
1-12 mo	20 (41.7)	28 (58.3)		
12-36 mo	28 (45.9)	33 (54.1)		
36-60 mo	13 (40.6)	19 (59.4)		
60-120 mo	14 (26.9)	38 (73.1)		
≥120 mo	6 (26.1)	17 (73.9)		
Duration of dialysis^§^ mo	18 (6, 24)	12 (6, 24)	*z* = −0.689	0.491

Abbreviations: BMI: body mass index; mo: month; min: minute. ^∗^Continuous variables conforming to the assumption of normal distribution and the assumption of homogeneity were compared using Student's *t*-tests. ^†^Pearson *χ*^2^ test or Fisher exact test was used to compare categorical variables. ^§^Ordinal data were compared using the Mann-Whitney *U* test. A *p* value of less than 0.05 was considered statistically significant.

**Table 2:** The baseline biochemical characteristics of the patients.

	Normal bone density (*n* = 81)	Low bone density (*n* = 135)	*t*/*z*	*p*
Creatinine^§^	109 (88.5, 135)	111 (90, 139)	*z* = −0.477	0.634
BUN^§^	6.38 (5.28, 7.215)	6.5 (5.02, 8.62)	*z* = −1.153	0.249
FBG^§^	5.1 (4.9, 5.5)	5.2 (4.9, 5.5)	*z* = −0.135	0.892
Calcium^§^	2.44 (2.37, 2.52)	2.42 (2.35, 2.52)	*z* = −0.352	0.725
Albumin^§^	47.4 (44.6, 48.7)	47 (44.9, 48.4)	*z* = −0.660	0.509
Phosphorus^§^	0.96 (0.83, 1.05)	1.03 (0.87, 1.11)	*z* = −2.190	0.029
CO2CP^§^	23.9 (22.3, 26.45)	23.8 (22, 25.7)	*z* = −0.538	0.591
iPTH^§^	77.83 (78.05, 111.15)	75.36 (52.95, 110.48)	*z* = 1.652	0.200
ALP^§^	68.5 (57, 91.75)	77 (63.5, 100)	*z* = −2.038	0.042
TC^§^	5.01 (4.495, 5.805)	5.1 (4.56, 5.77)	*z* = −0.569	0.569
TG^§^	1.44 (1.025, 1.7)	1.28 (1, 1.65)	*z* = −0.776	0.438
LDL-C^§^	2.53 (2.06, 3.01)	2.54 (2.08, 2.98)	*z* = −0.355	0.722
HDL-C^∗^	1.49 ± 0.37	1.53 ± 0.4	*t* = −0.763	0.446
P1NP^§^	55.59 ± 5 (40.25, 71.92)	61.81 (43.44, 88.89)	*z* = −1.843	0.065
*β*CTX^§^	0.68 (0.4, 0.92)	0.69 (0.47, 0.96)	*z* = −0.838	0.402
25(OH)D^§^	23.71 (17.9, 29.44)	22.19 (16.22, 28.85)	*z* = −0.716	0.474

Abbreviations: BUN: blood urea nitrogen; FBG: fasting blood glucose; CO2CP: carbon dioxide combining power; iPTH: serum intact parathyroid hormone; ALP: alkaline phosphatase; TC: total cholesterol; TG: triacylglycerol; LDL-C: low-density lipoprotein cholesterol; HDL-C: high-density lipoprotein cholesterol; P1NP: N-terminal propeptide of type 1 collagen; *β*-CTX: *β*-isomerized C-terminal telopeptide of type 1 collagen; 25(OH)D: 25-hydroxyvitamin D3. ^∗^Continuous variables conforming to the assumption of normal distribution and the assumption of homogeneity were compared using Student's *t*-tests. ^§^Ordinal data were compared using the Mann-Whitney *U* test. A *p* value of less than 0.05 was considered statistically significant.

**Table 3:** The use of immunosuppressive drugs in the patients.

	Normal bone density (*n* = 81)	Low bone density (*n* = 135)	*t*/*χ*^2^/*z*	*p*
Duration of glucocorticoid^§^	26 (9, 52)	41 (15, 88)	*z* = −2.492	0.013
Current dose of glucocorticoid^∗^	8.76 ± 2.31	8.36 ± 2.56	*t* = 1.382	0.241
Cumulative of glucocorticoid^§^	10435 (5580, 17510)	14060 (7335, 26190)	*z* = −2.265	0.025
Average daily glucocorticoid dose^§^	12.905 (10.79, 18.02)	11.509 (9.78, 15.28)	*z* = 0.643	0.423
Duration of tacrolimus^§^	22 (6, 42.5)	23 (3, 60)	*z* = −0.14	0.886
Duration of cyclosporine^§^	0 (0, 0)	0 (0, 0)	*z* = −2.150	0.032
Duration of MMF^§^	26 (8.5, 50)	38 (12, 88)	*z* = −2.168	0.030

Abbreviations: MMF: mycophenolate mofetil. ^∗^Continuous variables conforming to the assumption of normal distribution and the assumption of homogeneity were compared using Student's *t*-tests. ^§^Ordinal data were compared using the Mann-Whitney *U* test. A *p* value of less than 0.05 was considered statistically significant.

**Table 4:** Binary logistic regression analysis of bone mineral density.

	B	S.E.	P	OR	OR 95% CI	
					Lower	Upper
BMI	-0.149	0.055	0.007	.0862	0.773	0.960
Weekly exercise sessions	-0.335	0.170	0.049	0.715	0.512	0.998
Phosphorus	2.447	0.970	0.012	11.552	1.725	77.354
ALP	0.016	0.006	0.006	1.016	1.005	1.028
Duration of glucocorticoid use	0.011	0.004	0.007	1.011	1.003	1.018
Constant	-.236	1.717	.891	1.266		

Abbreviations: BMI: body mass index; ALP: alkaline phosphatase; B: regression coefficient; S.E.: standard error; OR: odds ratio; OR 95% CI (lower and upper): OR 95% confidence interval (lower and upper).

## Data Availability

Due to respect for and protection of patient privacy, the data generated and/or analyzed in this study are not publicly available. However, they are available from the corresponding author on reasonable request.
